# A 16-Channel Receive, Forced Current Excitation Dual-Transmit Coil for Breast Imaging at 7T

**DOI:** 10.1371/journal.pone.0113969

**Published:** 2014-11-24

**Authors:** Samantha By, Joseph V. Rispoli, Sergey Cheshkov, Ivan Dimitrov, Jiaming Cui, Stephen Seiler, Sally Goudreau, Craig Malloy, Steven M. Wright, Mary Preston McDougall

**Affiliations:** 1 Department of Biomedical Engineering, Texas A&M University, College Station, Texas, United States of America; 2 Advanced Imaging Research Center, University of Texas Southwestern Medical Center, Dallas, Texas, United States of America; 3 Department of Radiology, University of Texas Southwestern Medical Center, Dallas, Texas, United States of America; 4 Philips Medical Systems, Cleveland, Ohio, United States of America; 5 Department of Electrical and Computer Engineering, Texas A&M University, College Station, Texas, United States of America; 6 VA North Texas Health Care System, Dallas, Texas, United States of America; University Hospital Essen, Germany

## Abstract

***Purpose*:**

To enable high spatial and temporal breast imaging resolution via combined use of high field MRI, array coils, and forced current excitation (FCE) multi channel transmit.

***Materials and Methods*:**

A unilateral 16-channel receive array insert was designed for use in a transmit volume coil optimized for quadrature operation with dual-transmit RF shimming at 7T. Signal-to-noise ratio (SNR) maps, *g*-factor maps, and high spatial and temporal resolution *in vivo* images were acquired to demonstrate the utility of the coil architecture.

***Results*:**

The dual-transmit FCE coil provided homogeneous excitation and the array provided an increase in average SNR of 3.3 times (max 10.8, min 1.5) compared to the volume coil in transmit/receive mode. High resolution accelerated *in vivo* breast imaging demonstrated the ability to achieve isotropic spatial resolution of 0.5 mm within clinically relevant 90 s scan times, as well as the ability to perform 1.0 mm isotropic resolution imaging, 7 s per dynamics, with the use of bidirectional SENSE acceleration of up to R = 9.

***Conclusion*:**

The FCE design of the transmit coil easily accommodates the addition of a sixteen channel array coil. The improved spatial and temporal resolution provided by the high-field array coil with FCE dual-channel transmit will ultimately be beneficial in lesion detection and characterization.

## Introduction

Magnetic resonance imaging (MRI) has become a promising tool for improved diagnosis and evaluation of breast cancer, owing to its ability to provide high sensitivity and resolution [1]–[4]. To accurately detect and characterize breast lesions using MRI, in particular for dynamic contrast enhancement (DCE) studies [5], high temporal resolution is required in addition to high spatial resolution [6], [7]. At standard clinical fields (3T and below), however, the practicality of simultaneous high spatial and temporal resolution is limited by the achievable signal-to-noise ratio (SNR) [8], [9]. In order to explore the potential of recently available commercial high field scanners to address this limitation, several groups are investigating breast imaging and spectroscopy at 7T [8], [10]–[17]. The increase in sensitivity provided by high fields can be exploited to increase spatial and/or temporal resolution. In addition, it has been shown that further temporal acceleration can be achieved at high fields from enhanced SENSE performance due to the decrease in coil sensitivity degeneracy [18].

Despite these advantages, translation of high field MRI to standard clinical usage for breast (or any other) studies has not been straightforward, primarily due to the increased Larmor frequency of hydrogen and the associated wavelength effects that confound homogeneity and power deposition [10], [19]–[22]. Thus, much of the work in breast imaging has focused on optimizing the RF coil design [23], [24]. Unilateral and bilateral breast receive-only and transmit/receive array coils with up to 30 elements have been presented, working in concert with a variety of transmit coil designs [25]–[29]. We have previously reported the advantages of transmitting with “forced current excitation” (FCE) in the design of a highly homogenous quadrature volume breast coil for 7T [30]. This uniform B_1_ excitation provides predictable imaging contrast and consistent fat suppression. In this work we describe a unilateral 16-channel receive array insert designed for use in the FCE transmit volume coil being driven for optimized quadrature operation with dual-transmit RF shimming. The performance of the new coil was evaluated based on its signal-to-noise ratio (SNR) advantage over the volume coil and its ability to enable high spatial and high temporal resolution *in vivo* breast images. The improved resolution afforded by this synergistic design is expected to greatly benefit breast lesion detection and characterization.

## Methods

### Dual Transmit Coil

**Figure 1:**
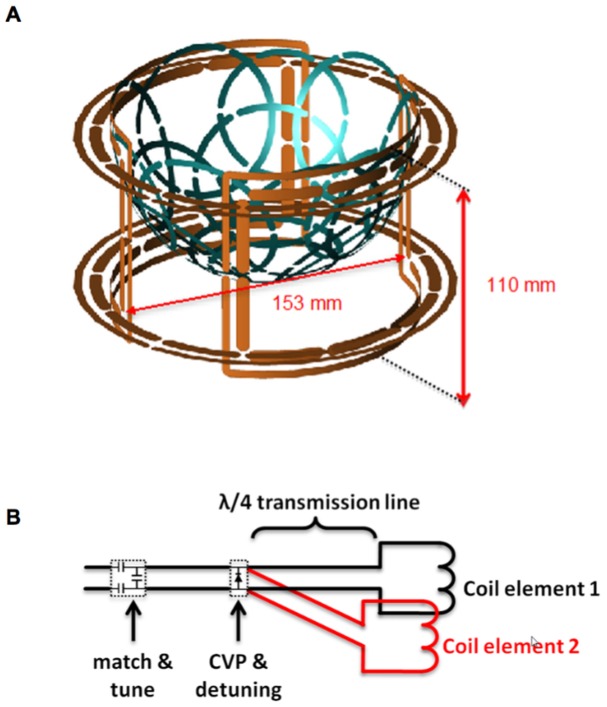
Overview of transmit and receive coil setup. A) Rendering of transmit coil (in orange, i.d. = 153 mm, depth = 110 mm) with 16-channel receive array insert (in blue), and B) Schematic overview of FCE detuning circuitry utilizing λ/4 transmission lines to open-circuit each transmit coil element when diodes at the common voltage point (CVP) are biased.

All hardware was designed and tested for use on a whole-body 7T scanner configured with two independent transmit channels (Achieva, Philips Medical Systems). The transmit coil was based on a previously-reported unilateral Helmholtz-saddle configuration designed to produce homogenous quadrature excitation of the pendant breast using forced current excitation (FCE) [30]. A rendering of the volume coil with the array in place is shown in Fig. 1A. In this design, a Helmholtz pair and a saddle pair are orthogonal to each other, and the two elements of each pair are connected to a common voltage point (CVP) with quarter wavelengths of transmission line. This forces equal currents to be delivered to the top and bottom loops of the Helmholtz pair, as well as to the left and right elements in the saddle pair. Each pair was separately driven by one of the independent transmit channels. As previously reported, the resulting transmit field was highly homogenous, despite the asymmetric loading in the anterior-posterior direction presented by the thorax when imaging the pendant breast and, more significantly, in the presence of the asymmetric loading presented by the hemispherical array insert. The Helmholtz loops had an inner diameter of 160 mm and were separated by 80 mm, and the saddle elements were constructed on a cylindrical tube with an inner diameter of 153 mm and centered inside of the Helmholtz pair. The saddle elements each were segmented by twelve breaks with 13 pF capacitors (1111C series, Passive Plus) and the Helmholtz elements each with twenty breaks, alternating between 10 and 12 pF capacitors (1111C series, Passive Plus) after the breaks for the feed points. All four transmit elements had individual co-planar shields.

The FCE configuration also enabled straightforward detuning of the transmit coil during receive. As illustrated in Fig. 1B, a shunt PIN diode (UM9415, Microsemi) rated for 1000 W was placed at the CVP such that when forward-biased during receive, the quarter wavelength lines presented open circuits to the transmit coil elements. As a potential advantage, this approach creates physical separation between the DC control lines and the field-of-view.

### Receive Array

A dedicated 16-channel receive array was designed for insertion in the open-sided FCE dual transmit volume coil. The array was constructed on a polycarbonate hemispherical shell fabricated with a fusion deposition modeling (FDM) rapid prototyping machine. The shell had an inner diameter of 142 mm and a thickness of 3 mm, providing a usable volume of approximately 750 mL, such that the geometrical filling factor would be 52%, 64%, 79%, and 95% of the array volume for average breast sizes 36A, B, C, and D, respectively [31]. This ensures usability of the array on a large portion of the patient population, while keeping the geometrical filling factor at more than 50% in most cases. As pointed out by other authors [6], in custom-designed close-fitting coils, as compared to the commercially available open-form biopsy breast coils, the proximity to the sample is one of the major contributors to improvements in the achievable SNR.

**Figure 2:**
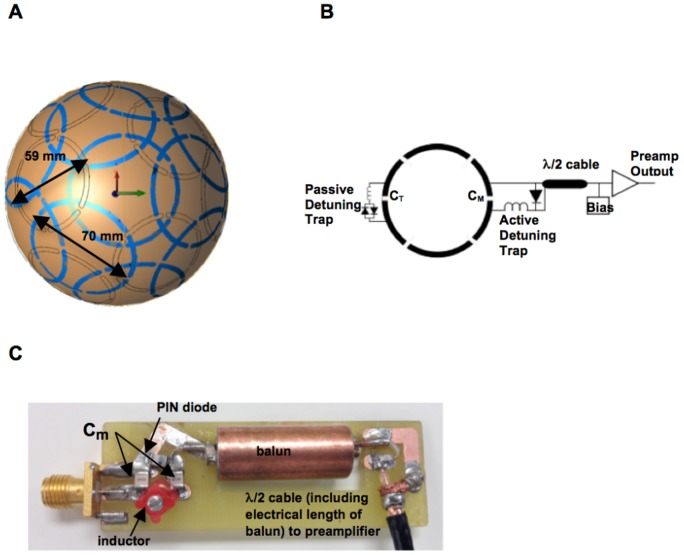
16-channel unilateral breast receive array. A) Layout of overlapped receive elements (as observed from the bottom of the hemisphere), highlighting each element's position and size (70 mm loops in blue, 59 mm loops in gray). There are 3 rows of coils in the anterior-posterior direction, each row having 1, 6, or 9 coils, respectively. B) Circuit schematic of a single receive element including preamplifier chain. Each element is segmented by six breaks, with a passive and active detuning trap around the tune and match capacitors, respectively. C) Detachable board including the active detuning trap, balun, and cable connection to 16-channel interface box.

Element size and placement on the shell were determined by arranging pentagonal and hexagonal tiles in the “soccer ball geometry” presented by Wiggins et al. [32]. The resulting layout consisted of ten coils with an inner diameter of 70 mm and six coils with an inner diameter of 59 mm in an overlapping coil arrangement over three rows in the anterior-posterior (A/P) axis, as shown in Fig. 2A. Roughly speaking there were four independent coils elements in either the left-to-right (L/R) or the foot-to-head (F/H) direction.

All elements were fabricated from 0.2 mm thick copper-clad FR-4 printed circuit board (PCB). Each loop was etched as a “C”-shape with an opening that, when pulled together, resulted in a loop conformed to the surface of the hemispherical shell. Each element had six breaks. As shown in Fig. 2B, fixed capacitors (9.1 pF and 11 pF, 1111C series, Passive Plus) were placed across four breaks, and a variable tuning capacitor (46MN series, Passive Plus) was placed across the break opposite the feed. Crossed diodes (UM9989B, Microsemi) and a variable inductor (164 series, Coilcraft) in parallel with the tuning capacitor formed a passive trap to increase isolation of each element during transmit [33].

**Figure 3:**
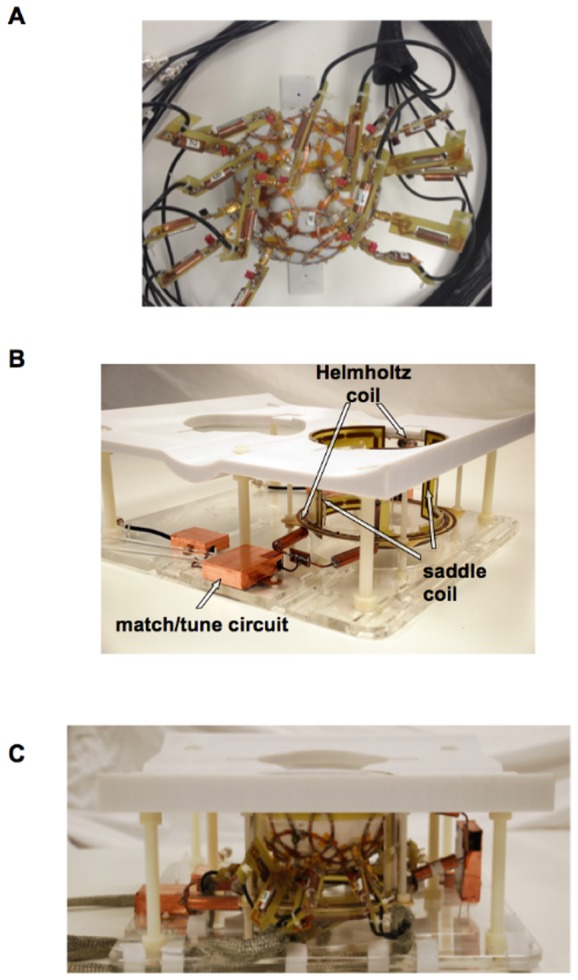
Photographs of the coil. A) the 16-channel receive array with external boards connected as viewed from the bottom of the hemisphere, B) the dual-transmit FCE volume coil showing one of the common voltage points and C) the final configuration with the receive array inside the transmit coil and patient support structure.

Given the limited clearance and fit inside the transmit coil, the required circuitry at the element feed points was located on detachable circuit boards and connected after inserting the array in the transmit volume coil. These boards contained the active trap configuration (described further below), a balun, and a half-wavelength coaxial cable (including the electrical length of the balun) that connected to the Philips 16-channel receiver interface box, which housed the isolating preamplifiers and bias control. An SMA plug connected to the feed point of the element allowed for direct connection to an end-launch SMA receptacle on the detachable board. A labeled photograph of one of the 16 boards is shown in Fig. 2C. The balun was constructed from a user-tunable prefabricated inductor can (3T-CC-3T, Correct Coil). The active trap consisted of a PIN diode (MA4P7470F-1072, M/A-COM), a variable inductor (164 series, Coilcraft), and a fixed match capacitor (13–33 pF, 1111C series, Passive Plus). Since the external board included the match capacitor, disconnecting the board from the loop open-circuited the corresponding receive element; thus, removing all but one board created an isolated setting for tuning a single element. Once an individual element was matched and tuned to 50 Ω, a capacitor was removed from the coil in order to tune the active trap on a non-resonant structure. With the diode forward biased with an external 100 mA source, an inductor probe was used to detect the resonant frequency of the trap and the variable inductor was adjusted until the trap was tuned to 298 MHz. The isolation provided by the trap for each coil element was measured as the change in the S_12_ measurement between two decoupled, shielded probes when the PIN diode was unbiased and biased. Isolation was greater than 20 dB in all cases. The 16 cables were bundled in groups and cable traps were created for each bundle to control any remaining induced currents on the cable shields. The cable bundles could be easily routed through alternative paths in the space under the coil platform and this allowed for either left or right side breast imaging. Photographs of the final assembly of the transmit volume coil and the 16-channel receive array with the external boards attached are shown in Fig. 3.

### Imaging

All phantom and *in vivo* imaging described below was performed on a Philips Achieva 7.0T research system.

#### Phantom imaging

A homogenous phantom was used to illustrate the performance of the 16-channel receive array insert in comparison to receiving with the FCE volume coil in T/R mode (with the array removed to eliminate potential effects of the array on the volume coil performance). The phantom was constructed as a hemispheric shell to maximally fill the 750 mL array volume. The phantom was filled with canola oil to mimic the lipid properties of the breast [34]. SNR was evaluated using data acquired from two scans with identical imaging parameters, with the RF and gradient amplifiers turned on in one scan and off in the other to generate a noise-only scan [35]. Imaging for the purpose of evaluating SNR was performed using typical parameters for the widely used clinical sequence of sagittal 3D T1-weighted fast gradient echo multishot pulse sequence (THRIVE). While the clinical THRIVE sequence uses fat suppression, phantom SNR imaging was performed with the fat saturation turned off since the phantom was entirely composed of canola oil. The following scan parameters were used: TR = 4.0 ms, TE = 1.77 ms, flip angle = 8°, resolution of 1×1×2 mm^3^ (4 mm-thick overcontiguous slices), bandwidth of 1056 Hz/pixel, acquisition matrix 160×160.

In addition, we have previously reported marked improvements in homogeneity when sample-specific dual-transmit optimization was used in order to achieve circular polarization [36], [37]. Specifically, regions of low signal intensity and decreased SNR were observed if the relative excitation phase between the Helmholtz and the saddle pairs was not optimized for that particular sample. Given the variability in size and composition of the human breast, we expect this per-subject optimization of the quadrature field to play an important role in achieving optimum image quality. Therefore, the Helmholtz pair and the saddle pair were driven in parallel by two independent 4 kW RF amplifiers. The procedure used to calibrate the channels was described previously [37] and it was applied for each individual phantom scan and on a per-volunteer basis. Briefly, B_1_ calibration maps were collected, per individual transmit channel, using a dual-TR 3D B_1_ acquisition [38] with nominal flip angle of 50°, TR1/TR2 = 35/140 ms, and resolution 2×2×10 mm^3^. Both magnitude and phase data were acquired and used to set the relative transmit channel amplitude and phase to achieve true circular polarization over a user-selected region in the middle slice of the 3D field maps. Volume selective power optimization [39] was also used in the same region to achieve final 90° calibration. CLEAR (constant level of appearance) homogeneity correction was turned off and SENSE was set to one (i.e. no acceleration). The SNR map was calculated by dividing, on a pixel-per-pixel basis, the signal from the phantom image by the standard deviation of a 30 mm×30 mm area at the same location in the noise-only scan [29]. As is customary, the noise-only scans from the individual 16 elements were also used to generate a noise correlation matrix for the array. Geometry factor (*g*-factor) maps were obtained with the same imaging protocol, adding SENSE acceleration factors of 1×, 2×, or 3× in the foot-to-head (F/H) direction combined with acceleration of 1×, 2×, or 3× in the left-to-right (L/R) direction. The *g*-factor maps were calculated using the manufacturer's on-the-scanner reconstruction algorithm. In the analysis of these maps, we report the mean *g*-factor over the entire phantom using the middle slice of the 3D volumetric acquisition, as well as the maximum measured *g*-factor in the same slice.

#### In vivo imaging

Imaging for this specific study was performed under a protocol approved by the University of Texas Southwestern Medical Center Institutional Review Board and after obtaining written informed consent. A healthy 40-year-old volunteer and a 32-year-old patient with a fibroadenoma were recruited for this study. The subjects were in prone position with the head resting on pillows and with arms either to the side or above the head. An *in vivo* comparison of the SNR of the 16-channel receive insert versus the standalone FCE volume T/R coil was performed using a 3D T1-weighted THRIVE sequence similar to the one used in the phantom study. Total scan time was 1∶32 minutes. To investigate the *in vivo* SENSE performance of the coil as quantified by *g*-factor mapping, images were also obtained with and without various accelerations up to 9× (1×, 2×, 3× in the L/R and F/H directions) with the 16-channel receive array insert.

Fat suppressed 3D T1-weighted sagittal breast imaging was performed by fixing the total acquisition time at the clinically relevant 90 s and two different spatial resolutions were obtained: (i) 1-mm isotropic resolution, which is commonly used clinically, and (ii) high resolution 0.5-mm isotropic images (i.e. an 8-fold volumetric reduction as compared to the clinically-used protocol), with SENSE acceleration factors of 2.2 in the F/H direction and 2.1 in the L/R directions. The dual-transmit settings of the coil were calibrated per individual patient, as described in the phantom imaging section.

The 1-mm resolution images obtained for the spatial resolution comparison were then obtained with 3-fold acceleration in both L/R and F/H directions, reducing acquisition time from 90 to 7 s. This constituted a 13-fold temporal acceleration as compared to the standard clinical dynamic resolution. Other relevant acquisition parameters, used in both the spatial and temporal comparison studies, were as follows: TR = 4 ms, TE = 2 ms, flip angle = 8°, FOV = 160×160×160 mm, SPAIR TI = 150 ms.

Lastly, high spatial and temporal resolution images were acquired in a patient diagnosed with a fibroadenoma (up to 3.5 cm in size, located 3 cm from the nipple) in the right breast. Images were acquired using a 0.6-mm isotropic THRIVE (i.e. 5-fold volumetric reduction as compared to the 1-mm clinical protocol), with bidirectional SENSE of 2×2 [(F/H)×(L/R), in a total acquisition time of 60 s (i.e. 1.5-fold temporal acceleration)].

## Results and Discussion

### SNR

**Figure 4:**
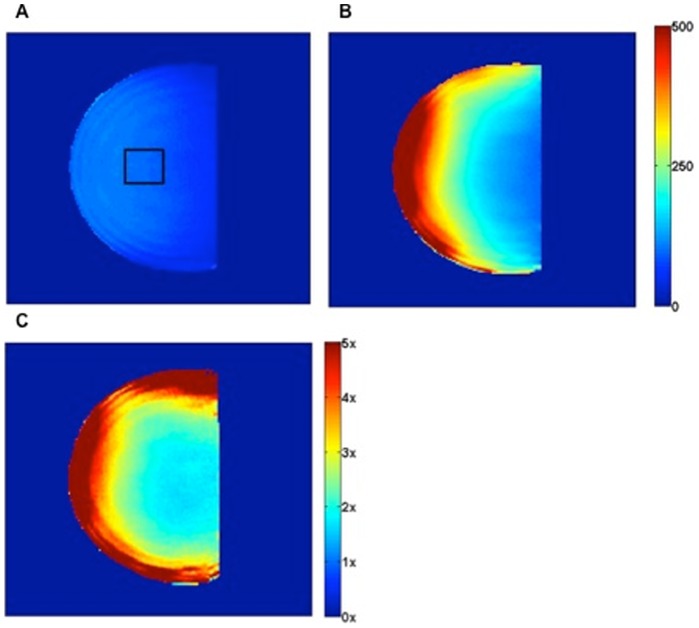
Comparison of SNR maps in a phantom between the 16-channel receive array and volume coil. Improvements in SNR when using close-fitting 16-channel array. SNR maps acquired with A) the FCE volume coil alone and B) the 16-channel receive array. The sagittal view through the middle of a hemispherical homogenous canola oil phantom is shown (a.u. SNR). C) SNR ratio between the 16-channel receive array and the volume coil demonstrates a mean SNR improvement of a factor of 3.3 over the entire area of the phantom, with a mean SNR gain of 2.1× in the middle of the phantom marked by the black ROI in (A). The periphery of the phantom experiences a local high (up to 10-fold) increase in SNR.

One of the primary motivations for using ultra-high field imaging is the inherent increase in detection sensitivity. To illustrate the gains in sensitivity achieved with the array coil, SNR maps of the homogenous phantom are shown in Fig. 4, acquired with the standalone FCE volume coil with the array removed (Fig. 4A) and with the 16-channel receive array coil (Fig. 4B). The array improved SNR throughout the entire phantom, achieving a mean SNR improvement of 3.3 times with up to a 10.8 times increase (Fig. 4C) at the edges. As expected, the array sensitivity steadily decreased with distance into the phantom [40], and a 2.1 times increase in SNR was achieved in a region of interest in the center of the phantom towards what would be the chest wall. While the homogeneity of the FCE transmit coil was unaffected by the presence of the array, there were other practical effects due to the addition of the conducting elements and components. As would be expected, the addition of the array required the transmit coil to be re-tuned, though not out of the range of the variable capacitor. In addition, based on the Philips' driving scales, a 22% increase in power was required to achieve a 90-degree flip angle.

### Noise correlation and g-factor

**Figure 5:**
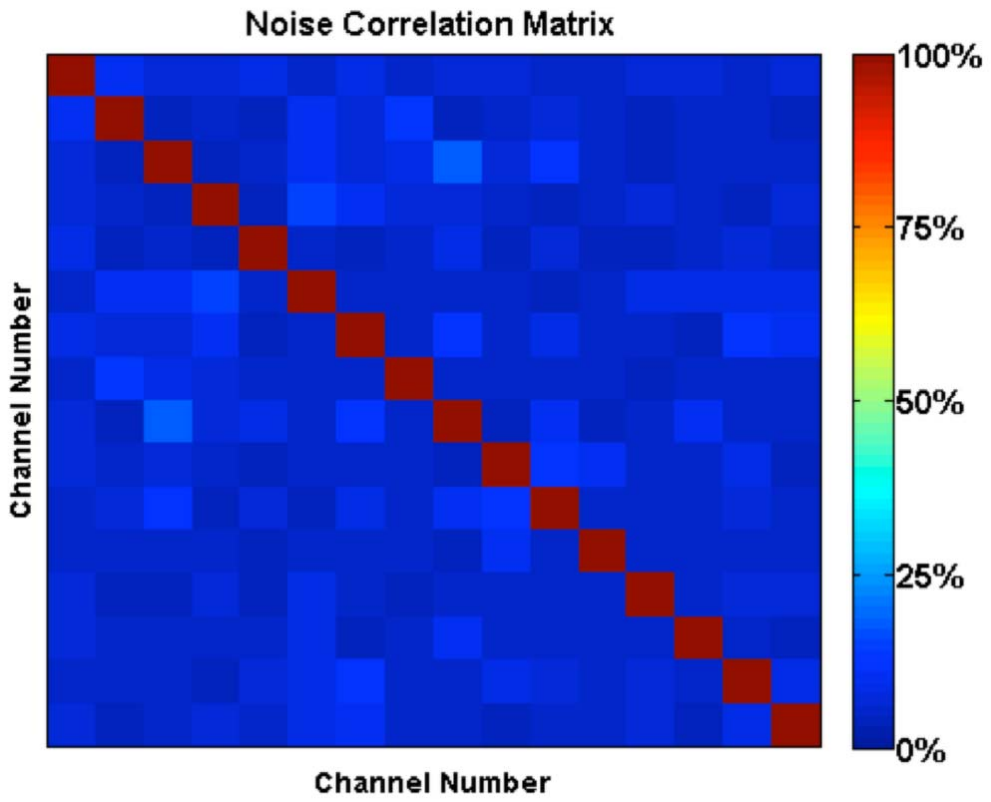
Noise correlation matrix of the 16 channel receive elements. Noise correlation matrix from the 16-channel receive array acquired with the uniform phantom. The mean correlated value is 6.6%, with a minimum of 3.6% and a maximum of 17.7%.

The 16×16 noise correlation matrix is shown in Fig. 5, with a range from 3.6% to 17.7% and a mean correlated value of 6.6% in the off-diagonal elements of the matrix. It is worth noting that this compares favorably to the performances of other unilateral breast arrays designed for 7T, which have reported mean values ranging from 3.9–10% and maximum values ranging from 25–44% [27], [29], while recognizing that our particular low values could be due in part to the minimal loading properties of the canola oil.

**Figure 6:**
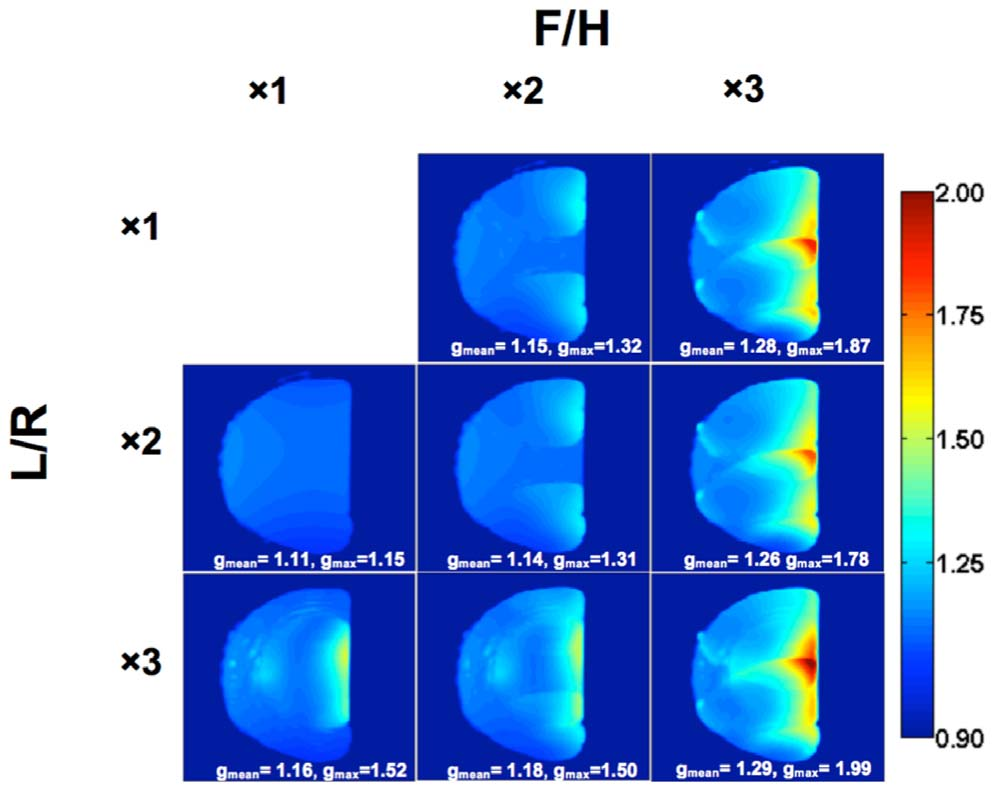
*g*-factor maps of a sagittal mid-slice in a phantom. SENSE acceleration was applied in the left-to-right (L/R) and foot-to-head (F/H) directions using acceleration factors of 1×, 2×, or 3× in each direction. Mean and maximum values are reported for each acceleration.

Figure 6 displays the average and maximum *g*-factor values and *g*-factor distribution for up to a nine-fold (F/H×L/R = 3×3) reduction factor as calculated from the homogenous phantom data. It is generally accepted that an SNR loss of up to 20% (*g*-factor 1.2) is considered acceptable [41], and this condition was maintained for total accelerations through six, corresponding to a reduction in imaging time from 24.2 to 5.8 s.

### In vivo imaging

**Figure 7:**
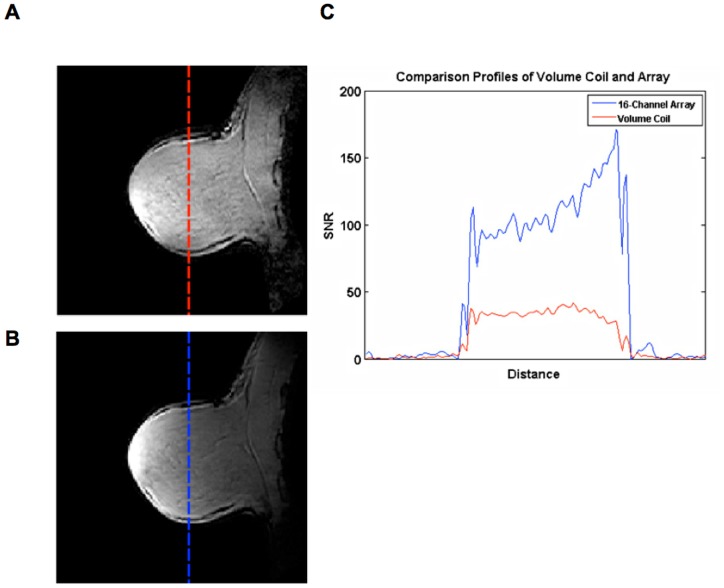
In vivo SNR profiles. Images of the right breast sagittal slice from a volunteer obtained with A) the FCE coil in T/R mode, B) the transmit FCE coil with the 16-channel receive array insert (different windowing was used compared to (A) due to the very high SNR values close to the array elements) and C) a comparison of the respective profiles. The *in vivo* results demonstrate comparable SNR gains to the phantom data; there is approximately a 3.5× improvement in mean SNR throughout the breast.

**Figure 8:**
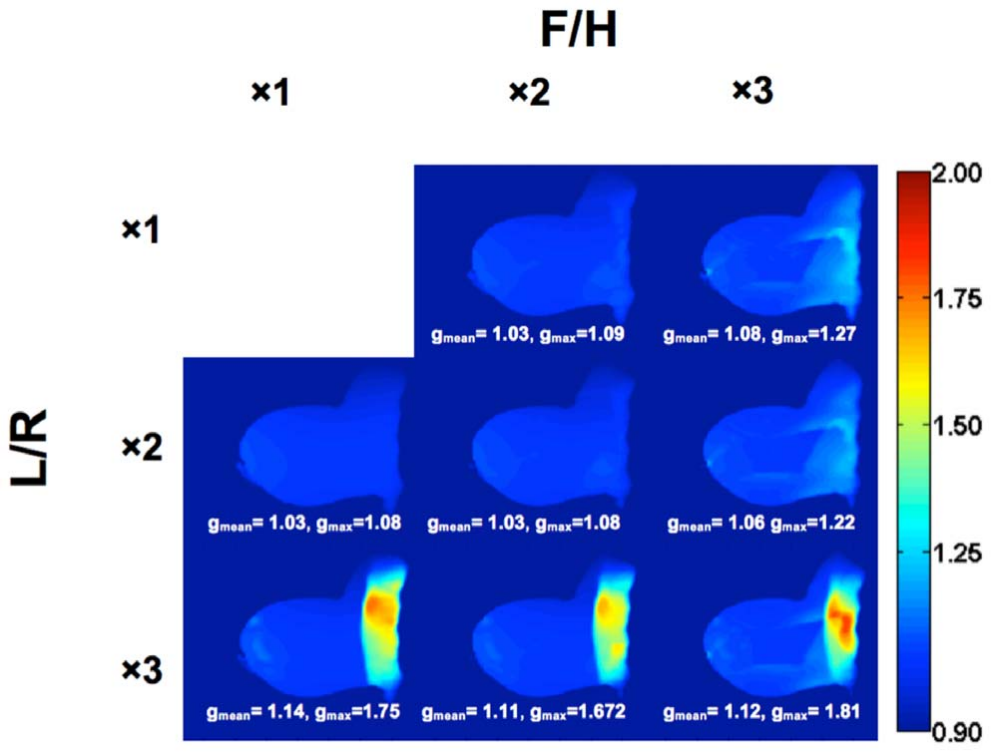
*g*-factor maps of a sagittal mid-slice *in vivo*. SENSE acceleration was applied in the left-to-right (L/R) and foot-to-head (F/H) directions using acceleration factors of 1×, 2×, or 3× in each direction. With R = 4 (2×2), the mean and the maximum *g*-factor were 1.03 and 1.08, respectively, allowing for acquisition of just 25% of the k-space data. With the low *g*-factors, there should be negligible propagation of artifacts in the final reconstructed image.

Figure 7 compares the SNR of *in vivo* images obtained from a healthy volunteer with the 16-channel receive array and with the standalone FCE volume coil in T/R mode. A profile from the same slice of both images is extracted to highlight the increase in SNR with the 16-channel receive array, which provided a 3.5 times increase in mean *in vivo* SNR throughout the breast, agreeing well with the phantom data. In addition, the *in vivo* images show the penetration into the chest wall that the phantom imaging could/did not. It would be expected that the degree of penetration, however, will be dependent on the positioning and size of the subject. Figure 8 characterizes the *g*-factor mapping of the 16-channel receive array for reduction factors up to nine. It is evident that the receive array can enable accelerated imaging with negligible consequences up to R = 4, keeping the maximum *g*-factor under 1.08 even into the chest wall, which is out of the direct hemispherical FOV of the array.

**Figure 9:**
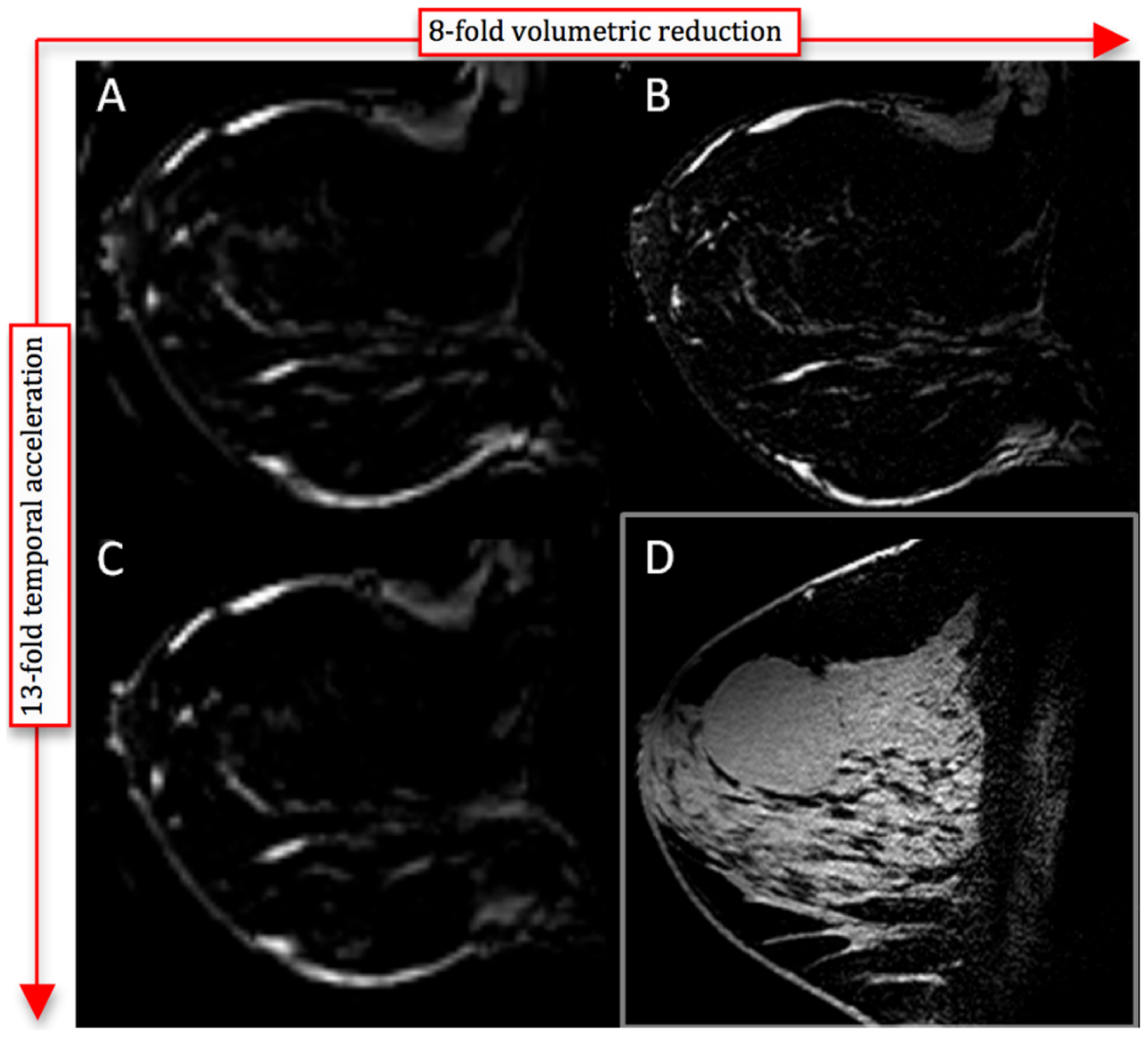
Increased spatial/temporal resolution in *in vivo* breast imaging (3D T1-weighted THRIVE with SPAIR fat suppression). A) Clinically standard 1-mm isotropic resolution with 90 s acquisition time from the same volunteer as Figs. 7 and 8; B) Higher spatial resolution: SENSE accelerated acquisition (2.2 F/H, 2.1 L/R) with 0.5 mm isotropic resolution with 90 s acquisition time, same volunteer as in (A); C) Higher temporal resolution: Ultrafast 7 s acquisition time with 1-mm isotropic resolution with SENSE factor 3×3 [F/H×L/R], same volunteer as in (A); D) High resolution image of a fibroadenoma (0.6-mm isotropic, SENSE 2×2, 60 s acquisition) in the right breast of a patient.

Breast images of the same healthy volunteer acquired with the array are shown in Fig. 9 A–C to illustrate the relevance of the increased SNR provided by the array with respect to providing spatial and temporal resolution improvements. The quality of the fat suppression is notable. An image with clinically standard spatial resolution (1 mm isotropic) and temporal resolution (90 s acquisition) is shown in Fig. 9A for comparison to images fixing acquisition time and improving resolution (Fig. 9B) and fixing resolution and decreasing acquisition time (Fig. 9C). The image in Fig. 9B was acquired with 0.5 mm isotropic resolution and SENSE acceleration factors of 2.2 in the F/H direction and 2.1 in the L/R directions to hold the acquisition time fixed at 90 s. This provides an 8-fold volumetric reduction as compared to the clinically used protocol and clearly shows improved tissue margins and morphological breast duct detail as compared to Fig. 9A while still maintaining sufficient SNR. The image in Fig. 9C was acquired with 1 mm isotropic resolution in 7 s using 3×3 SENSE acceleration with no visible degradation of the image quality as compared to Fig. 9A. This ultrafast acquisition time (13-fold temporal acceleration) lends itself well to possible future application in high-speed breast DCE MRI [5]. Finally, the clinical utility of the breast array is illustrated in Fig. 9D, showing an image of a breast fibroadenoma acquired with acceleration of two in both directions leading to 5-fold volumetric reduction as compared to the 1-mm clinical protocol, in a total acquisition time of 60 s (i.e. 1.5-fold temporal acceleration). The lesion is clearly delineated from the glandular tissue with no SENSE artifacts observable.

The immediate future direction of this work is the extension to bilateral imaging to increase the clinical utility of the described coils. On the transmit side, FCE lends itself well to bilateral implementation as the approach is insensitive to the coupling potentially presented by the addition of an adjacent transmit coil [42]. From a receive standpoint, since most 7T scanners are currently equipped with 32-channel receivers, translation to a bilateral design should be straightforward and doubling the number of channels on this configuration should provide additional improvements in the overall clinical performance.

## Conclusions

In summary, this paper has presented the significant improvements achieved by using a 16-channel receive array insert in a forced current excitation dual-transmit volume coil for 7T breast imaging. By using the array insert, a mean SNR improvement of 3.3 times was achieved in phantom imaging as compared to the closely-fitting volume coil in T/R mode. Unique properties of the FCE coil allowed the insert to be added with minimal modification to the transmit coil for detuning. In addition, the combined breast coil design benefits from the uniform B_1_ field produced by the FCE transmit coil, while the two independent transmit channels allowed for adjusting of the excitation to “true” quadrature mode for increased efficiency. Clinically, the SNR improvement provided by the receive array enabled the ability to perform bidirectional acceleration up to R = 9 without significant penalty, allowing for shorter acquisition times and improved spatial resolution. The high SENSE acceleration factors achieved demonstrated once again the synergy between high static field and parallel imaging, based on the advantage of the more inhomogeneous receive patterns exhibited at high fields [18]. The results clearly demonstrate the promise of coil design and optimization to enable effective MR imaging of the breast at 7T.
